# Waist circumference and cardiometabolic parameters in people of African/Caribbean ancestry with HIV in South London (CKD-AFRICA study)

**DOI:** 10.1177/09564624241233036

**Published:** 2024-02-20

**Authors:** Laura Cechin, Lourdes Dominguez-Dominguez, Lucy Campbell, Lisa Hamzah, Julie Fox, Royce P Vincent, Georgios K Dimitriadis, Louise Goff, Frank A Post

**Affiliations:** 1 8948 King’s College Hospital NHS Foundation Trust, London, UK; 2 4616 King’s College London, London, UK; 3 4968 St George’s Hospital NHS Foundation Trust, London, UK; 4 Guys and St Thomas’s NHS Foundation Trust, London, UK; 5 Synnovis Analytics, London, UK; 6 Leicester General Hospital, 4488 University of Leicester, Leicester, UK

**Keywords:** Metabolic syndrome, insulin resistance, waist circumference, HIV, black ethnicity

## Abstract

**Background:**

There are no validated waist circumference (WC) cut-offs to define metabolic syndrome in Black people with HIV.

**Methods:**

Cross-sectional analyses within the CKD-AFRICA study. We used Pearson correlation coefficients and receiver operating characteristic (ROC) curves to describe the relationship between WC and cardiometabolic parameters including triglycerides, cholesterol, glucose, glycated haemoglobin (HbA1c), and homeostatic model assessment for insulin resistance (HOMA-IR), and to identify optimal WC cut-offs for each of these outcomes.

**Results:**

We included 383 participants (55% female, median age 52 years) with generally well controlled HIV. Female and male participants had similar WC (median 98 vs. 97 cm, *p* = .16). Generally weak correlations (r^2^ < 0.2) between WC and other cardiometabolic parameters were observed, with low (<0.7) areas under the ROC curves. The optimal WC cut-offs for constituents of the metabolic syndrome, HbA1c and HOMA-IR ranged from 92 to 101 cm in women and 89-98 cm in men, respectively; these cut-offs had variable sensitivity (52%–100%) and generally poor specificity (28%–72%).

**Conclusions:**

In this cohort of Black people with HIV, WC cut-offs for cardiometabolic risk factors in male participants were in line with the recommended value of 94 cm while in female participants they vastly exceeded the recommended 80 cm for white women.

## Introduction

The metabolic syndrome (MetS) is a cluster of cardiovascular disease (CVD) risk factors encompassing dyslipidaemia, hypertension, hyperglycaemia, and central obesity.^1^ Waist circumference (WC) is an indicator of visceral fat, an independent risk factor for CVD alongside other factors like triglycerides (TG) and insulin resistance. WC cut-offs to define central obesity are population-specific; for example, WC thresholds of 94 and 80 cm are used for men and women of European ancestry while lower cut-offs are used for South Asian populations.^1^ A lack of validated cut-offs for people of African ancestry has led to a recommendation to use European WC cut-offs for these populations,^1^ even though the relationship between WC and visceral adipose tissue (VAT) differs substantially between black and white people.^2,3^ The appropriateness of using cut-offs derived in white populations for defining MetS in people of African ancestry has been questioned, in African Americans as well as sub-Saharan Africans.^4–7^

In people with HIV, introduction of antiretroviral therapy (ART) is generally associated with weight gain, and ART regimens that do not contain tenofovir disoproxil fumarate or efavirenz have been associated with greater weight increases.^8^ Although the metabolic consequences of ART-associated weight gain are still to be fully elucidated, increases in visceral adipose tissue have been reported, with women of African ancestry disproportionally affected.^9,10^ Hence, WC cut-offs to define MetS derived in general Black populations may not be applicable to those with well-controlled HIV on ART.

Few studies have examined the relationship between WC and other MetS/CVD risk factors (dyslipidaemia, hypertension, hyperglycaemia, insulin resistance) in people of African ancestry with HIV.^11^ We sought to identify optimal WC cut-offs for constituents of the MetS in people of African Ancestry with HIV in South London.

## Methods

Our analyses were conducted in the Cardiovascular disease, Kidney disease, and Diabetes in People of African Ancestry with HIV (CKD-AFRICA) cohort, a cross-sectional sub-study of comorbidities and social determinants of health within the Genetic Determinants of Kidney Disease in People of African Ancestry with HIV (GEN-AFRICA) cohort (NCT05685810). The GEN-AFRICA cohort comprises over 3,000 individuals aged 18 years and over with HIV of (self-identified) Black ethnicities who were enrolled between 2018 and 2020 at 15 sites in the UK. For the CKD-AFRICA study, consecutive GEN-AFRICA participants aged 30 to 65 years were invited to attend a single study visit after an overnight fast at one of three South London HIV clinics. WC and three standardised blood pressure (BP) measurements were taken, and fasting serum triglyceride, high-density lipoprotein (HDL)-cholesterol, and glucose concentrations were measured. Questionnaires were used to collect information on prior diagnoses of hypertension (HPT), diabetes mellitus (DM), cardiovascular disease, and medications to treat these conditions. Blood samples were also collected for HbA1c and insulin analysis. Biochemical analysis was carried out in the UKAS ISO 15189:2012 accredited Synnovis laboratory at King’s College Hospital. The study was approved by a National Health Service Research Ethics Committee (20/LO/0946) and the Health Research Authority (IRAS 278244), and all participants provided informed consent.^12^

We used the joint interim statement criteria (JIS) for MetS (triglycerides >1.7 mmol/L; HDL-cholesterol <1.0 mmol/L in men and <1.3 mmol/L in women; BP ≥130/85 mmHg, or use of antihypertensive medication; glucose ≥5.6 mmol/L, or use of hypoglycaemic medications; and WC ≥94 cm in men and ≥80 cm in women) as reference.^1^ The homeostatic model assessment for insulin resistance (HOMA-IR) calculator v2.2.4 was used to evaluate insulin resistance^13^; the upper quartile of HOMA-IR in participants with FBG ≤5.6 mmol/L in the absence of hypoglycaemic medications was used to define insulin resistance. The CKD-EPI 2021 equation was used to estimate the glomerular filtration rate (eGFR).^14^ Analyses were restricted to participants who did not have severe comorbid disease; those with chronic kidney disease (eGFR <45 mL/min/1.73 m^3^), cardiac failure or liver cirrhosis, or a kidney or liver transplant) and those with type 1 DM were excluded; participants with type 2 DM on insulin were included in the main analyses but excluded from the insulin resistance analyses.

### Statistical analysis

Participant characteristics were described by sex and compared using Chi-squared test (or Fisher’s exact test for variables with frequency <5 in either subgroup) for categorical data, or Kruskal–Wallis test for continuous data. We evaluated the relationships between WC (exposure) and components of the MetS (triglycerides, HDL-C, FPG, systolic and diastolic BP), glycated haemoglobin (HbA1c), and HOMA-IR (outcomes) in women and men separately.

We graphically explored the relationship between WC and each outcome; participants on antihypertensives and diabetic medications were excluded from the analyses of BP and fasting glucose/HbA1c respectively, and those on insulin from the analysis of HOMA-IR. Pearson correlation coefficients were generated for each of the outcomes. The optimal WC cut-off for each of the outcomes was evaluated using receiver operating characteristic (ROC) curves with Youden indices. All statistical analyses were performed using R (R Foundation, Vienna, Austria; version 4.2.1).

## Results

A total of 398 participants were enrolled between September 2020 and January 2022, of whom 15 were excluded for severe comorbidities or type 1 DM. The median age of the 383 study participants was 52 (inter-quartile range [IQR] 45-57) years, 55% were women, and most had well controlled HIV infection (HIV RNA <200 copies/mL in 94%, HIV RNA <20 copies/mL in 83%; Table 1). The median BMI was 30.0 (IQR 26.6-34.2) kg/m^2^, and 65% of participants had systemic hypertension, 25% kidney disease, 18% diabetes mellitus (treatments displayed in Table S1), and 7% cardiovascular disease; 13% were on a statin. The female participants were younger and had higher BMI and HDL-cholesterol and lower systolic and diastolic BP, triglyceride and glucose measurements than men; 9.2% of female and 20.4% of male participants had triglycerides in the MetS range, and 30.1% of female and 12.8% of male participants had HDL-cholesterol measurements in the MetS range. HbA1c, insulin, HOMA-IR and WC measurements did not differ by sex. Most female participants (91%) had a WC ≥80 cm, and 64% of male participants had a WC ≥94 cm. Using these established WC cut-offs for white populations, 25% of women and 32% of men met the JIS criteria for MetS.

**Table 1.** Demographic and clinical characteristics of the CKD-AFRICA study participants.

*DEMOGRAPHICS/HIV PARAMETERS*	Overall (*N* = 383)	Female (*N* = 210)	Male (*N* = 173)	*p* value^a^
Age	52 [45, 57]	**51 [45, 56]**	**54 [45, 58]**	**0.040**
Region of birth				**<0.001**
Sub-Saharan Africa	275 (71.8)	167 (79.5)	108 (62.4)	
Caribbean	37 (9.7)	10 (4.8)	27 (15.6)	
UK/Other	71 (18.5)	33 (15.7)	38 (22.0)	
Time since HIV infection diagnosis (years)	14 [9, 18]	14 [9, 18]	14 [9, 18]	0.74
On antiretroviral therapy	380 (99.2)	207 (98.6)	173 (100.0)	0.32
Non-nucleoside reverse-transcriptase inhibitor (NNRTI)	134 (35.3)	81 (39.1)	53 (30.6)	0.11
Protease inhibitor (PI)	107 (28.2)	55 (26.6)	52 (30.1)	0.52
Integrase strand-transfer inhibitor (INSTI)	139 (36.6)	71 (34.3)	68 (39.3)	0.37
Nucleoside reverse-transcriptase inhibitor (NRTI)				
TDF/FTC	138 (36.3)	71 (34.3)	67 (38.7)	0.43
TAF/FTC	79 (20.8)	**33 (15.9)**	**46 (26.6)**	**0.016**
Other	163 (42.9)	**103 (49.8)**	**60 (34.7)**	**0.004**
Time since starting ART (years)	10 [7, 15]	10 [7, 15]	10 [6, 15]	0.79
HIV RNA <200 copies/mL	360 (94.0)	199 (94.8)	165 (93.1)	0.63
Nadir CD4 cell count	162 [69, 275]	137 [52, 265]	180 [83, 283]	0.09
Recent CD4 cell count	538 [372, 747]	544 [364, 750]	534 [383, 742]	0.87
HCV Ab positive	4 (1.1)	2 (1.0)	2 (1.2)	1.00
HBsAg positive	25 (6.9)	17 (8.5)	8 (4.9)	0.25
*OTHER CLINICAL PARAMETERS*				
Current smoker	36 (9.8)	17 (8.4)	19 (11.4)	0.43
BMI (kg/m^2^)	30.0 [26.6, 34.2]	**32.3 [27.8, 36.8]**	**28.2 [25.7, 31.2]**	**<0.001**
Systemic hypertension^c^	248 (65.1)	131 (62.7)	117 (68.0)	0.33
Diabetes mellitus^d^	69 (18.0)	32 (15.2)	37 (21.4)	0.154
Kidney disease^e^	97 (25.3)	53 (25.2)	44 (25.4)	1.00
Cardiovascular disease^f^	27 (7.0)	15 (7.1)	12 (6.9)	1.00
*METABOLIC SYNDROME PARAMETERS*				
Waist circumference (cm)	98 [90, 105]	98 [90, 107]	97 [90, 104]	0.162
Fasting HDL-cholesterol (mmol/L)	1.50 [1.20, 1.72]	**1.60 [1.30, 1.90]**	**1.30 [1.10, 1.58]**	**<0.001**
Fasting triglycerides (mmol/L)	1.00 [0.70, 1.40]	**0.90 [0.70, 1.22]**	**1.11 [0.77, 1.60]**	**<0.001**
Mean systolic blood pressure (mmHg)	128 [118, 144]	**125 [114, 140]**	**132 [121, 146]**	**0.002**
Mean diastolic blood pressure (mmHg)	83 [76, 91]	**83 [76, 89]**	**84 [77, 94]**	**0.07**
Fasting glucose (mmol/L)	5.0 [4.6, 5.5]	**4.9 [4.5, 5.3]**	**5.2 [4.7, 5.6]**	**<0.001**
*OTHER METABOLIC PARAMETERS*				
HbA1c (%)	5.7 [5.4, 6.1]	5.7 [5.4, 6.0]	5.7 [5.4, 6.2]	0.29
Fasting insulin (pmol/L)^b^	53.4 [26.9, 90.8]	56.4 [29.4, 91.1]	47.4 [24.0, 90.6]	0.38
HOMA-IR score^b^	1.02 [0.51, 1.73]	1.08 [0.57, 1.76]	0.93 [0.47, 1.70]	0.34

^a^Categorical data are described with absolute (n) and relative (%) frequencies and compared with the Chi-squared test (with continuity correction), except HCV Ab frequencies which are compared by Fisher’s exact test. Continuous data are described with the median and interquartile range and compared with the Kruskal–Wallis test

^b^Excludes participants receiving insulin.

^c^Systemic hypertension was defined by BP >130/85 and/or use of antihypertensive medications;

^d^diabetes mellitus by HbA1c >6.5% and/or use of hypoglycaemic medications;

^e^kidney disease by eGFR <60 mL/min/1.73 m^2^ or albumin/creatinine ratio >3 mg/mmol;

^f^cardiovascular disease by a history of ischaemic heart disease, congestive cardiac failure, or stroke.

HCV Ab: hepatitis C virus antibody. HBsAg: hepatitis B virus surface antigen. BMI: body mass index. HDL: high-density lipoprotein. HOMA-IR: Homeostasis model assessment for insulin resistance, based on fasting insulin levels. HOMA-B: Homeostasis model assessment for β-cell function, based on fasting insulin levels.

The linear regression models showed, in both women and men, generally weak correlations (r^2^ < 0.2) between WC and the other components of the MetS (Figure S1). A similarly weak correlation between WC and HbA1c was observed, particularly among female participants. The strongest correlation was observed for HOMA-IR, in both women (r^2^ 0.24, *p* < .0001) and men (r^2^ 0.25, *p* < .0017).

Results from the ROC models are shown in Table 2. All MetS constituents displayed low (<0.7) areas under the ROC curve, indicating that WC had poor discriminatory value. The optimal WC cut-offs for the other components of the MetS in women ranged from 92 to 101 cm, and in men from 89 to 98 cm, with variable sensitivity and generally poor specificity.

**Table 2.** Identification of optimal waist circumference cut-off values for the diagnosis of metabolic syndrome, diabetes mellitus and insulin resistance in female and male CKD-AFRICA participants.

	Area under ROC curve	Waist circumference cut-off (cm)	Sensitivity	Specificity
Female population				
HDL-cholesterol <1.3 mmol/L	0.51 (0.42, 0.61)	100 (71, 126)	0.52 (0.37, 0.65)	0.55 (0.46, 0.62)
Triglycerides >1.7 mmol/L	0.66 (0.56, 0.76)	96 (93, 105)	0.95 (0.68, 1.00)	0.43 (0.28, 0.73)
Systolic BP ≥130mmHg (or on anti-HPT meds)	0.58 (0.50, 0.66)	92 (84, 118)	0.82 (0.18, 0.93)	0.38 (0.22, 0.96)
Diastolic BP ≥85mmHg (or on anti-HPT meds)	0.59 (0.51, 0.67)	101 (87, 121)	0.58 (0.15, 0.90)	0.63 (0.27, 0.99)
Fasting glucose ≥5.6 mmol/L (or DM)	0.66 (0.59, 0.74)	92 (89, 103)	0.96 (0.90, 1.00)	0.33 (0.26, 0.40)
HbA1c ≥ 6.5% (or DM)	0.70 (0.62, 0.78)	100 (93, 102)	0.87 (0.74, 1.00)	0.58 (0.32, 0.69)
HOMA-IR ≥1.5	0.65 (0.57, 0.74)	100 (98, 104)	0.69 (0.52, 0.83)	0.64 (0.50, 0.77)
HOMA-IR ≥1.5 (excluding participants on oral anti-DM meds)	0.64 (0.55, 0.73)	100 (97, 113)	0.66 (0.30, 0.83)	0.65 (0.47, 0.93)
Male population				
HDL-cholesterol <1.0 mmol/L	0.57 (0.47-0.68)	89 (88, 102)	1.00 (0.64, 1.00)	0.28 (0.21, 0.35)
Triglycerides >1.7 mmol/L	0.54 (0.43-0.65)	93 (85, 115)	0.80 (0.20, 0.94)	0.38 (0.20, 0.93)
Systolic BP ≥130mmHg (or on anti-HPT meds)	0.66 (0.57-0.74)	97 (91 103)	0.62 (0.42, 0.82)	0.71 (0.46, 0.86)
Diastolic BP ≥85mmHg (or on anti-HPT meds)	0.65 (0.57, 0.74)	97 (93, 103)	0.60 (0.41, 0.81)	0.72 (0.47, 0.87)
Fasting glucose ≥5.6 mmol/L (or DM)	0.57 (0.48, 0.67)	98 (85, 115)	0.59 (0.20, 0.92)	0.63 (0.23, 0.94)
HbA1c ≥ 6.5% (or DM)	0.65 (0.55, 0.75)	98 (90, 106)	0.68 (0.38, 0.92)	0.66 (0.30, 0.86)
HOMA-IR ≥1.5	0.64 (0.54, 0.73)	96 (92, 115)	0.77 (0.26, 0.90)	0.53 (0.35, 0.97)
HOMA-IR ≥1.5 (excluding participants on oral anti-DM meds)	0.63 (0.53, 0.73)	96 (91, 120)	0.77 (0.23, 0.91)	0.54 (0.34, 0.98)

Data are reported with 95% confidence intervals. HDL: high-density lipoprotein; BP: blood pressure; HPT: hypertension; DM: diabetes mellitus; HOMA-IR: Homeostasis Model Assessment for Insulin Resistance.

The optimal WC cut-offs for HbA1c and HOMA-IR were 100 and 96-98 cm for women and men, respectively, with variable sensitivity (66%–87%) and generally poor specificity (53%–66%). For comparison, sensitivity, and specificity of the established WC cut-offs for white populations are presented in Table S2; while the WC of ≥80 cm for women was highly sensitive for the various cardiometabolic parameters, specificity was poor, ranging from 10%–12%. A sensitivity analysis of FPG restricted to participants without DM showed areas under the ROC curve and WC cut-offs of 0.60 (95%CI: 0.48, 0.70) and 92 (95%CI: 87, 110) cm for women, and 0.53 (0.41, 0.69) and 94 cm (79, 104) for men.

## Discussion

In this cohort of older people of African ancestry with longstanding, generally well-controlled HIV, we report a high prevalence of CVD risk factors including obesity, hypertension, and diabetes mellitus. While the identified WC cut-offs for cardiometabolic risk factors in our male participants were in line with the recommended value of 94 cm, the recommended 80 cm cut-off for women was exceeded in >90% of our female participants, and a WC cut-off of 92–101 cm may be more appropriate for cardio-metabolic risk factors in women of African ancestry with HIV, and a WC cut-off of 100 cm for insulin resistance. However, these WC cut-offs do not inherently establish values for diagnosing metabolic syndrome, and the low sensitivity and specificity question the clinical utility of WC cut-offs for other cardiometabolic parameters in this population.

Two previous studies have analysed WC in relation to other cardiometabolic parameters in sub-Saharan African women; both were conducted in South African urban populations, and neither reported HIV status of the participants. Peer et al. studied 707 women with a mean BMI of 33 kg/m^2^ and, similar to us, identified optimal WC cut-offs of 95–100 cm, variable areas under the ROC curves (AUC, 0.57–0.78), and low sensitivity (53%–67%), and specificity (56%–65%).^4^ Crowther et al. studied 1251 women with a mean BMI of 31 kg/m^2^ and identified optimal WC cut-offs ranging from 79 cm for elevated triglycerides to 88–90 cm for low HDL-cholesterol, dysglycaemia, hypertension and insulin resistance, all with low AUC (<0.70), sensitivity (52%–72%), and specificity (30%–69%).^5^ A study of African women in the USA (mean BMI 28 kg/m^2^) identified an optimal WC cut-off of 96 cm for insulin resistance, with favourable ROC curve characteristics (AUC 0.81, sensitivity 67%, specificity 87%).^7^ Other studies reported optimal WC cut-offs of 86, 92 and 94 cm, respectively, for two or more components of the MetS in South African women (mean BMI 26 kg/m^2^),^6^ South African women with HIV (median BMI 28 kg/m^2^),^11^ and women of West-African ancestry (mean BMI 27 kg/m^2^).^15^

These studies corroborate that WC cut-offs for European women are not appropriate to define MetS in women of African ancestry irrespective of HIV status. The wide range in identified optimal WC cut-offs in these studies reflect the generally low sensitivity and specificity for cardiometabolic risk factors of such cut-offs. A poor correlation between WC and the dyslipidaemia components of the MetS in women of African ancestry is consistent with an altered relationship between WC/BMI and VAT; abdominal obesity in this population predominantly reflects subcutaneous adipose tissue (SAT) and is less commonly accompanied by excessive accumulation of visceral fat that drives dyslipidaemia.^2,7^ Moreover, the relationship between WC and insulin resistance in people with HIV may be affected by exposure to different ART regimens.^10,16^

Our study included a well-characterized cohort of people of African and Caribbean ethnicities living with HIV in the UK but was not intended to determine WC values to define metabolic syndrome in these populations. We acknowledge some limitations. We did not study people of these ethnicities without HIV, people with HIV of other ethnicities, or individuals with poorly controlled HIV. Both women and men were well represented, and while our study was particularly focused on somewhat older people, those aged 65 years and above, who may display a different relationship between WC and cardiometabolic parameters, were excluded. The use of statins may have affected the relationship between WC and HDL-cholesterol. We did not perform imaging which would have allowed differentiation between visceral and subcutaneous abdominal fat. Finally, the study was not designed nor powered to examine the effects of ART and specific antiretroviral medications.

In conclusion, we identified a WC of 92–101 cm, as compared to the currently recommended 80 cm cut-off, to be associated with cardiometabolic parameters in women of African ancestry with HIV. As in previous studies in sub-Saharan African general populations, poor correlation was observed between WC and other components of the MetS. Cardiovascular risk assessments based on age, gender, blood pressure, lipids and glycaemia status may be more informative in clinical practice than assessments to determine the presence or absence of MetS. The positive correlation between WC and adverse cardiometabolic parameters suggest that a reduction in WC, as may be achieved through lifestyle interventions, medical or surgical therapies, may serve as a clinically useful strategy to improve health outcomes in these populations.

## Supplemental Material

Supplemental Material - Waist circumference and cardiometabolic parameters in people of African/Caribbean ancestry with HIV in South London (CKD-AFRICA study) Supplemental Material for Waist circumference and cardiometabolic parameters in people of African/Caribbean ancestry with HIV in South London (CKD-AFRICA study) by Laura Cechin, Lourdez Dominguez-Dominguez, Lucy Campbell, Lisa Hamzah, Julie Fox, Royce P Vincent, Georgios K Dimitriadis, Louise Goff and Frank A Post in International Journal of STD & AIDS.

## Data Availability

The database contains personal and sensitive information and is therefore not publicly available. Access to the study data and/or samples is governed by the National Health Service data access policy and those of King’s College Hospital NHS Foundation Trust, the study sponsor. The GEN-AFRICA and CKD-AFRICA studies are open to collaboration, and all requests from researchers who meet the criteria for access to fully anonymized patient level data will be considered. Enquiries and requests can be submitted for review to the principal investigator (Prof. Frank Post; email: frank.post@kcl.ac.uk).
