# Supplementary material for: Waist circumference and cardiometabolic parameters in people of African/Caribbean ancestry with HIV in South London (CKD-AFRICA study)
Source: Int J STD AIDS. 2024 Feb 20;35(7):521–6. doi: 10.1177/09564624241233036 (PMC11157977; doi:10.1177/09564624241233036)
Supplement: Supplemental Material - Waist circumference and cardiometabolic parameters in people of African/Caribbean ancestry with HIV in South London (CKD-AFRICA study) [file sj-pdf-1-std-10.1177_09564624241233036.pdf]

Table S1: Medications prescribed for diabetes mellitus amongst the study participants.

	Overall	Male	Female	BMI <30kg/m^2	BMI ≥30kg/m^2
n	383	173	210	192	191
Biguanide: metformin	41 (10.7)	25 (14.5)	16 (7.6)	16 (8.3)	25 (13.1)
Sulfonylurea: Gliclazide	13 (3.4)	6 (3.5)	7 (3.3)	4 (2.1)	9 (4.7)
DPP-IVi: Sitagliptin	6 (1.6)	2 (1.2)	4 (1.9)	0 (0.0)	6 (3.1)
PPARγA: Pioglitazone	1 (0.3)	1 (0.6)	0 (0.0)	0 (0.0)	1 (0.5)
GLP1RA	3 (0.8)	2 (1.2)	1 (0.5)	1 (0.5)	2 (1.0)
Dulaglutide	2 (0.5)	2 (1.2)	0 (0.0)	1 (0.5)	1 (0.5)
Liraglutide	1 (0.3)	0 (0.0)	1 (0.5)	0 (0.0)	1 (0.5)
SGLT2i	3 (0.8)	3 (1.7)	0 (0.0)	1 (0.5)	2 (1.0)
Empagliflozin	2 (0.5)	2 (1.2)	0 (0.0)	1 (0.5)	1 (0.5)
Canagliflozin	1 (0.3)	1 (0.6)	0 (0.0)	0 (0.0)	1 (0.5)
Insulin	10 (2.6)	8 (4.6)	2 (1.0)	6 (3.1)	4 (2.1)

[1]Categorical data are described with absolute (n) and relative (%) frequencies and compared with the Chi-squared test (with continuity correction).

DPP-IVi: Dipeptidyl peptidase 4 inhibitor. PPARγA: Peroxisome proliferator-activated receptor-γ agonists. GLP1RA: Glucagon-like peptide-1 receptor agonist. SGLT2i: Sodium-glucose cotransporter inhibitor.

Table S2: Sensitivity and specificity of reference WC cut-offs for other MetS components, HbA1c and HOMA2-IR score.

	Sensitivity % (95%CI)	Specificity % (95%CI)
**Female population: WC reference cut-off ≥80 cm**		
HDL-cholesterol <1.3 mmol/L	91.3 (79.2, 97.6)	9.6 (5.5, 15.4)
Triglycerides >1.7 mmol/L	100 (82.4, 100)	10.2 (6.2, 15.4)
Systolic BP ≥130mmHg (or on anti-HPT meds)	93.0 (85.4, 97.4)	10.7 (5.8, 17.5)
Diastolic BP ≥85mmHg (or on anti-HPT meds)	92.9 (85.3, 97.4)	10.6 (5.8, 17.4)
Fasting glucose ≥5.6 mmol/L (or DM)	100 (92.8, 100)	11.7 (7.1, 17.8)
HbA1c ≥6.5% (or DM)	100 (88.8, 100)	10.7 (6.6, 16.2)
HOMA-IR ≥1.5	96.9 (89.2, 99.6)	12.0 (7.0, 18.8)
HOMA-IR ≥1.5 (excluding participants on oral anti-DM meds)	96.2 (87.0, 99.5)	12.4 (7.3, 19.4)
**Male population: WC reference cut-off ≥94 cm**		
HDL-cholesterol <1.0 mmol/L	77.3 (54.6, 92.2)	38.7 (30.8, 47.0)
Triglycerides >1.7 mmol/L	68.6 (50.7, 83.2)	38.0 (29.8, 46.6)
Systolic BP ≥130mmHg (or on anti-HPT meds)	72.0 (61.8, 80.9)	46.8 (35.5, 58.4)
Diastolic BP ≥85mmHg (or on anti-HPT meds)	70.1 (58.6, 80.0)	42.1 (32.0, 52.7)
Fasting glucose ≥5.6 mmol/L (or DM)	67.2 (54.0, 78.7)	38.5 (29.1, 48.5)
HbA1c ≥6.5% (or DM)	75.7 (58.8, 88.2)	39.7 (31.4, 48.5)
HOMA-IR ≥1.5	78.4 (64.7, 88.7)	42.9 (33.2, 52.9)
HOMA-IR ≥1.5 (excluding participants on oral anti-DM meds)	76.7 (61.4, 88.2)	44.1 (33.8, 54.8)

Figure S1: Correlation between waist circumference and other metabolic syndrome parameters (A), HbA1c and HOMA-IR (B)

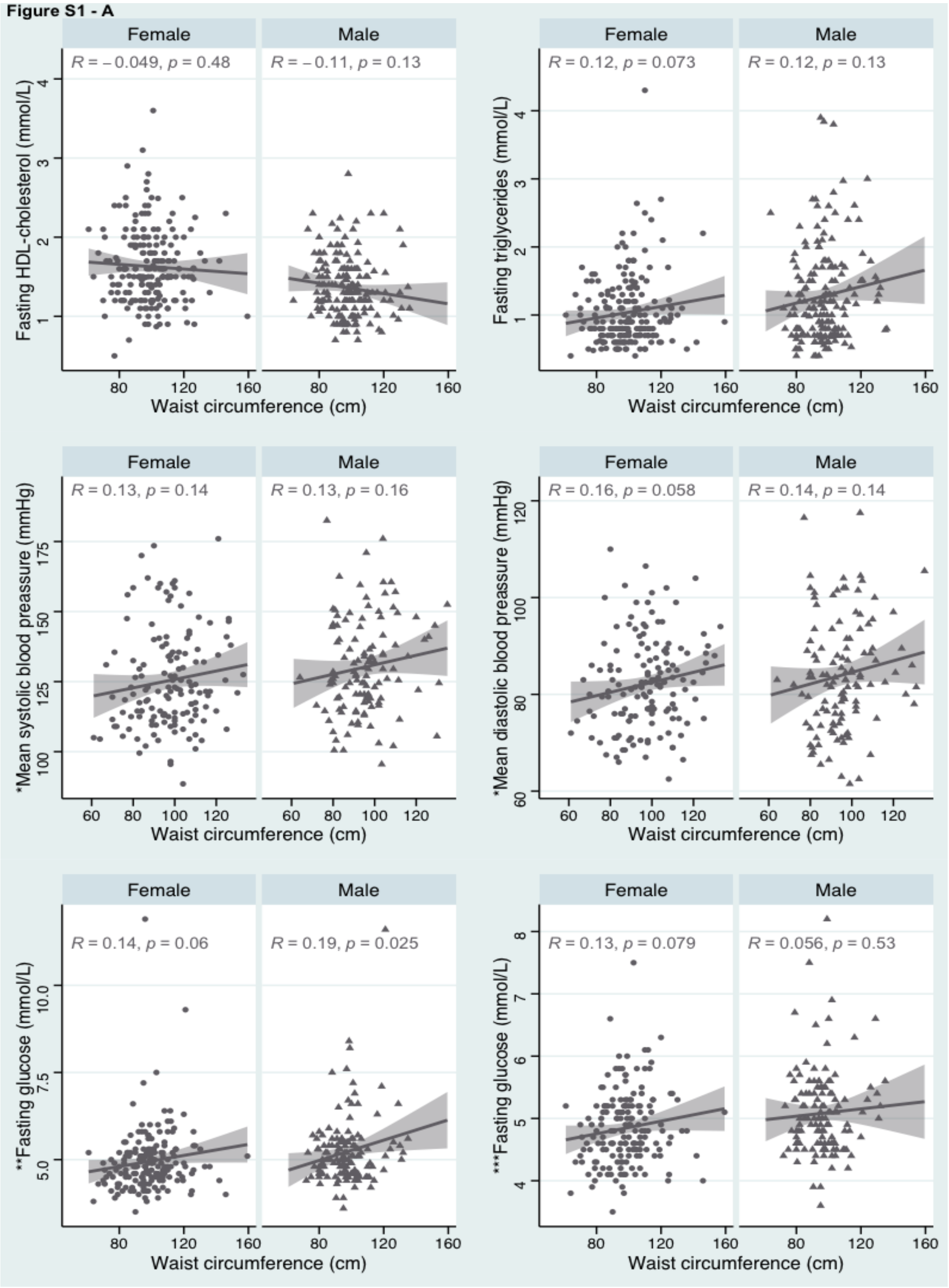

**Analysis of systolic and diastolic blood pressure excludes participants on anti-hypertensive medications;*
***Analysis of fasting glucose excludes participants on hypoglycaemic medications;*
****Analysis of fasting glucose excludes participants diagnosed with DM*

Figure S1 - B

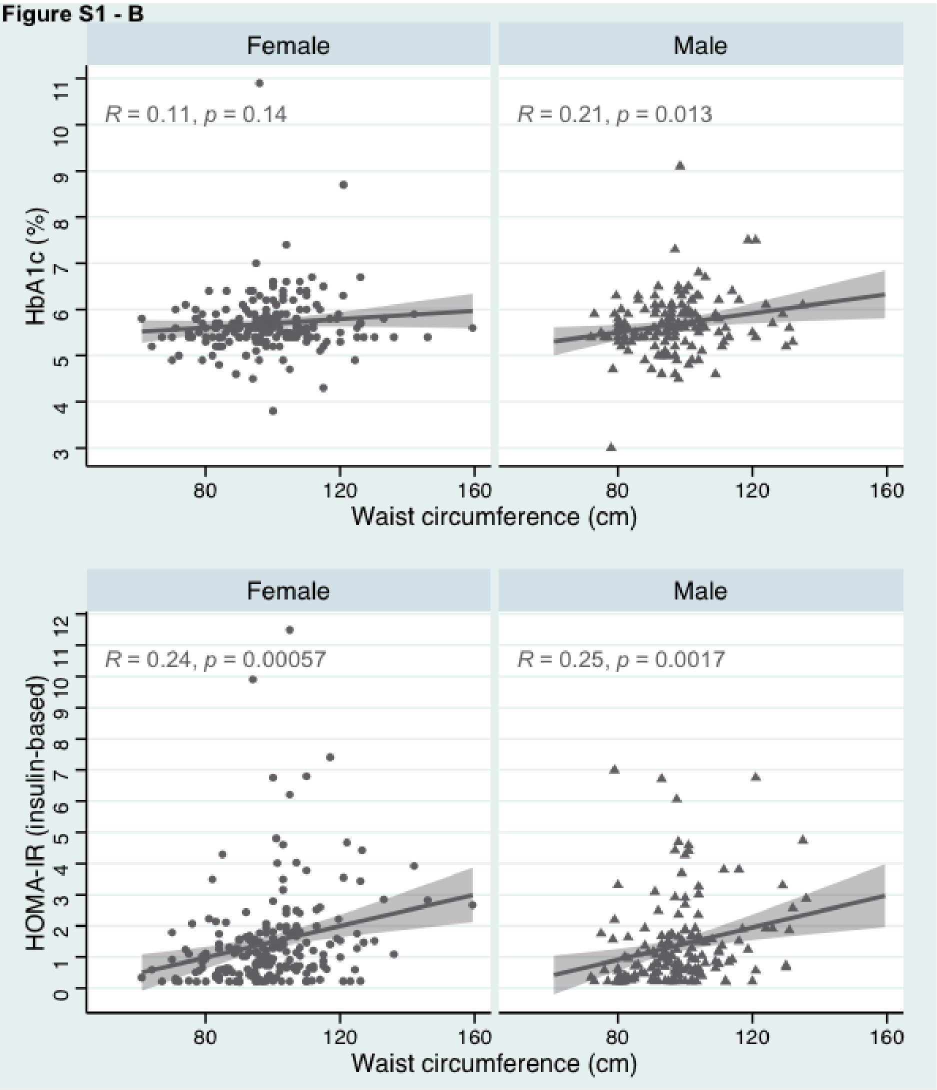

Analysis of HbA1c excludes participants on hypoglycaemic medications; analysis of HOMA-IR excludes participants on insulin
